# Analysis of the longitudinal stability of human plasma miRNAs and implications for disease biomarkers

**DOI:** 10.1038/s41598-024-52681-5

**Published:** 2024-01-25

**Authors:** Ursula S. Sandau, Jack T. Wiedrick, Trevor J. McFarland, Douglas R. Galasko, Zoe Fanning, Joseph F. Quinn, Julie A. Saugstad

**Affiliations:** 1 Department of Anesthesiology and Perioperative Medicine, Oregon Health and Science University, Portland, OR USA; 2 Biostatistics and Design Program, Oregon Health and Science University, Portland, OR USA; 3 Department of Neurosciences, University of California San Diego, La Jolla, CA USA; 4 Department of Neurology, Oregon Health and Science University, Portland, OR USA

**Keywords:** Diagnostic markers, Molecular neuroscience

## Abstract

There is great interest in developing clinical biomarker assays that can aid in non-invasive diagnosis and/or monitoring of human diseases, such as cancer, cardiovascular disease, and neurological diseases. Yet little is known about the longitudinal stability of miRNAs in human plasma. Here we assessed the intraindividual longitudinal stability of miRNAs in plasma from healthy human adults, and the impact of common factors (e.g., hemolysis, age) that may confound miRNA data. We collected blood by venipuncture biweekly over a 3-month period from 22 research participants who had fasted overnight, isolated total RNA, then performed miRNA qPCR. Filtering and normalization of the qPCR data revealed amplification of 134 miRNAs, 74 of which had high test–retest reliability and low percentage level drift, meaning they were stable in an individual over the 3-month time period. We also determined that, of nuisance factors, hemolysis and tobacco use have the greatest impact on miRNA levels and variance. These findings support that many miRNAs show intraindividual longitudinal stability in plasma from healthy human adults, including some reported as candidate biomarkers for Alzheimer’s disease.

## Introduction

Clinically approved biomarker assays are important paraclinical parameters that are commonly used to aid in the diagnosis of a number of diseases, such as Alzheimer’s disease (AD), multiple sclerosis, cancer, and infectious disease^1–4^. An ideal biomarker provides sensitive, specific, stable, and reliable measurements that are informative to the disease in question. Furthermore, biomarkers assayed in blood, urine, and saliva, which are collected by routine minimally- or non-invasive procedures, have the potential for greater clinically utility at reduced costs than less accessible biofluids, such as cerebrospinal fluid (CSF)^5^. Current clinically approved biomarkers lack some of these criteria, leaving clinical limitations such as an inability to longitudinally monitor disease progression and/or assess a therapeutic response.

Over the last decade, the use of circulating extracellular RNAs (exRNAs), especially microRNAs (miRNAs), as novel biomarkers for human disease has grown considerably^6^. The Extracellular RNA Communication Consortium (ERCC), funded through the National Institutes of Health (NIH) Common Fund, identified RNA biomarkers for nearly 30 diseases including AD, Parkinson’s disease, glioblastoma, vascular inflammation, cardiovascular disease, insulin resistance, and multiple sclerosis^7–16^. ExRNAs, including miRNAs, are found in virtually all biofluids^17–21^ where they are localized to protein particles (high-density lipoproteins or Argonaute proteins), extracellular vesicles (exosomes or microvesicles), and/or small nanoparticles (exomeres or supermeres)^22–25^.

MiRNAs are a class of small non-coding RNA that regulate post-transcriptional gene expression. Since changes in miRNA levels can occur in response to most if not all diseases, miRNAs have been a major focus of disease biomarker studies. In our previous ERCC studies, we discovered and then validated in two independent patient cohorts a set of CSF miRNAs that classify AD from neurologically normal control participants^14,26^, then showed that five of these miRNAs showed decreasing median expression levels across the ordered diagnosis of control to mild cognitive impairment (MCI) to AD^27^. These five miRNAs also work in combination to classify AD and MCI, and classification performance was further improved when the miRNAs were combined with CSF Aβ_1–42_ and tau ratio measurements^27^, demonstrating the potential of miRNAs to serve as longitudinal biomarkers for at least AD.

Changes in plasma and serum miRNA levels have also been identified in aging-associated diseases including cardiovascular disease, cancer, arthritis, dementia, cataract, osteoporosis, diabetes, hypertension, and neurodegenerative diseases^28^. Thus, there is substantial evidence supporting miRNA measurements in plasma as biomarkers for human diseases. However, to date, there have been few investigations into the longitudinal stability of miRNAs in plasma or serum from healthy humans^29–35^ and most are limited by participant sample size (n = 1–2 participants)^30,31,33^, number of longitudinal blood samples assayed (n = 2–3 draws)^29,30,34^, participants excluded based on potential confounders (e.g., tobacco use)^35^, and/or number of miRNAs assessed (< 20 miRNAs)^34,35^*.* One study did assess > 700 miRNAs in plasma samples collected from human research participants (n = 5 males and n = 5 females) at four time points (days 1, 2, 7, and 10) in either the morning or afternoon using qPCR. The overall conclusion was that miRNA levels were consistent between individuals and not significantly altered by sex, time of day, and sampling intervals less than 2 weeks^32^. To the best of our knowledge a longitudinal study investigating the stability of plasma miRNAs at regular intervals for an extended period of time has yet to be performed.

Here we examine the intraindividual longitudinal expression levels and stability of miRNAs in plasma over a 3-month period obtained biweekly from 22 human healthy control adults of moderate age. Stable miRNAs are preferred as biomarkers because they are reliably measurable at predictable levels in healthy individuals; consequently, perturbances in their levels in unhealthy individuals have enhanced diagnostic value. Further considerations to miRNA biomarkers are that technical variance in the assays over time may lead to inconsistency in measured miRNA levels, and biological variance due to fasting status, stress, and medications may also influence miRNA levels. To address these considerations, we developed a qPCR analysis pipeline that incorporates calibrations to adjust for exogenous sources of technical variance, as well as normalization to adjust for endogenous biological variance. This pipeline enables miRNAs to be scored along a quantitative stability index scale and examined for the confounding impact of common factors on measured miRNA levels and intraindividual variance.

## Results

### Participant demographics and hemolysis scores

We collected blood samples by venipuncture biweekly over a 3-month period from 22 human research participants, for a total anticipated 7 draws per individual. As a few participants missed a scheduled blood draw and/or samples were omitted due to assay technical errors, the final number of blood draws analyzed was 136 with the following distribution: n = 4 with 5 draws, n = 10 with 6 draws, and n = 8 with 7 draws (Supplementary Fig. 1). Our study cohort consisted of n = 15 females and n = 7 males, at ages between 25 to 65 years of age (Supplementary Fig. 2A). Participants fasted overnight for a majority of blood draws (Supplementary Fig. 2B). For hemolysis scores (see “Hemolysis proxy assessment” in “Methods”), we found an order-of-magnitude range from approximately 1–10, with approximately 25% of the draws showing appreciable hemolysis (score > 7). A large but not critical intensity (score ~ 6) was most typical across the 136 blood draws (Supplementary Fig. 3).

### MiRNA calibrator and normalizer considerations

**Figure 1:**
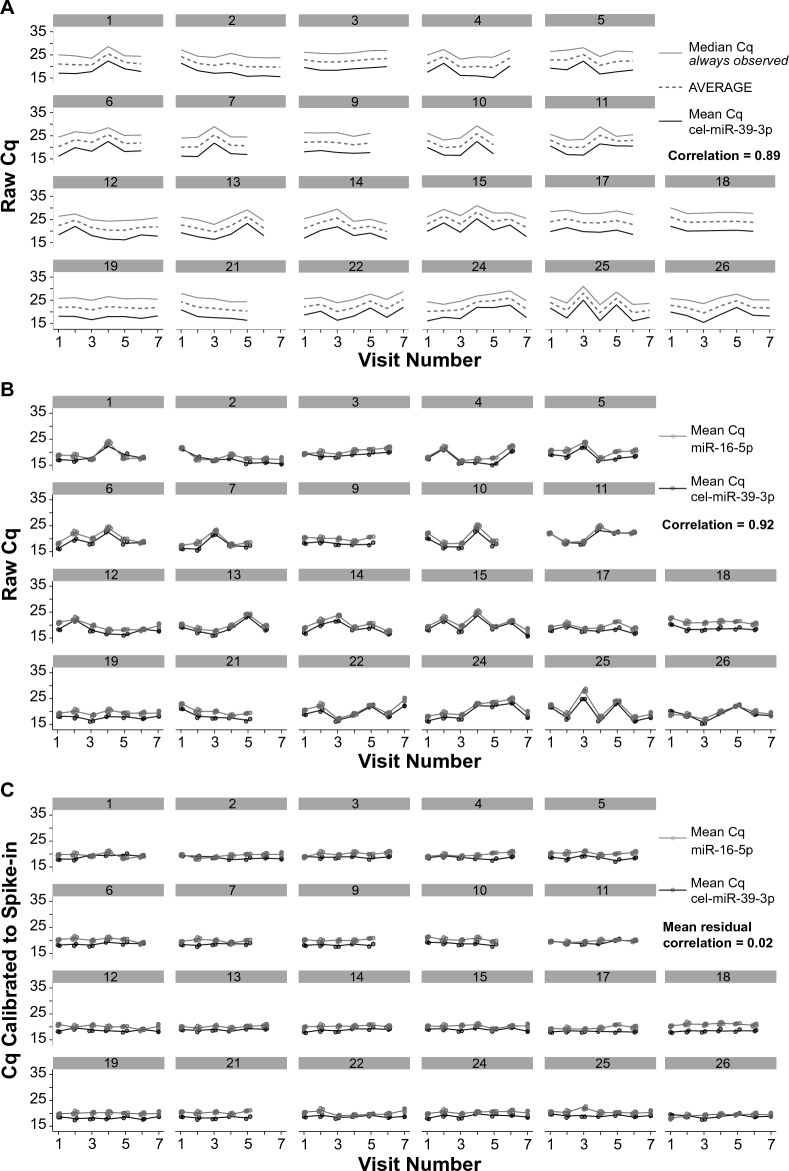
Calibration with the exogenous control cel-miR-39-3p corrects for a majority of technical assay variance. (**A**) Within each study participant (n = 22) (i) the median raw Cq values of the 36 always-observed miRNAs (solid grey line), (ii) mean raw Cq values for cel-miR-39-3p control (solid black line), and (iii) the average of the median Cq values of miRNAs always observed and cel-miR-39-3p (dashed grey line) are shown for each visit number. Spearman rank correlation was used to relate the median Cq values of the 36 miRNAs always observed to mean Cq values for cel-miR-39-3p (r = 0.89). (**B**) Within each study participant (i) the mean raw Cq values of miR-16-5p, the primary endogenous miRNA used for normalization (solid grey line) and (ii) mean raw Cq values for cel-miR-39-3p (solid black line) are shown for each visit number. Spearman rank correlation was used to relate the mean Cq values of miR-16-5p to those of cel-miR-39-3p (r = 0.92). (**C**) Within each study participant the raw Cq values were calibrated to cel-miR-39-3p values. The calibrated mean Cq values for miR-16-5p (solid grey line) and cel-miR-39-3p (solid black line) for each visit number are shown. The mean residual Spearman rank correlation (r = 0.02) is the correlation that remains after calibration is applied. In panels (**B**) and (**C**) the qPCR assay technical replicates for miR-16-5p and cel-miR-39-3p are depicted by the circles.

We first assessed the extent of assay variance on miRNA raw Cq values (not calibrated, not normalized). We identified a set of 36 miRNAs that are always observed within each participant, on each study visit date (Supplementary Table 1A, Mean % participant where always observed = 100%). The intraindividual median Cq value of these 36 miRNAs showed spikes in the data by visit (e.g., participant 1, visit number 4, Fig. 1A) that were not related to hemolysis of the sample but did correlate (r = 0.89) to the mean cel-miR-39-3p spike in control Cq values at the same visit, suggesting that this variance in the data was due to differences in RNA isolation and/or qPCR efficiency (Fig. 1A). Likewise, the mean Cq values for cel-miR-39-3p correlated (r = 0.92) with the mean Cq values of the primary endogenous control miRNA (miR-16-5p) for the same plasma sample on the same study visit date (Fig. 1B), suggesting that some but not all of the variance in measured miR-16-5p levels was due to the same cause. Thus, we used cel-miR-39-3p to calibrate the expression values of the miRNAs to correct for this technical assay variance. The cel-39-3p calibration dramatically increased consistency across repeated measurements for the same donor and delinked the endogenous variation from the technical variation, as evidenced by the low mean residual correlation with miR-16-5p (r = 0.02 is the correlation that remains after calibration is applied) (Fig. 1C). To further examine the relative magnitudes of sources of assay variance we calculated technical residuals for all miRNAs by subtracting out the mean level within each miRNA and the mean level within each participant, and then plotted (i) the raw Cq residuals of all 134 miRNAs included in the stability and metadata analysis (see Supplementary Table 1A for list of 134 miRNAs), followed by the Cq values resulting from each sequential step of (ii) cel-39-3p calibration, (iii) process batch calibration, and (iv) endogenous normalization (Supplementary Fig. 4). After calibrating all plasma samples by cel-39-3p, very little residual variation remained and subsequent calibration for batch effects (e.g., the order that the plasma samples were processed for qPCR) had negligible impact on Cq values. Thus, nearly all remaining differences in the miRNA level was attributed to changes within a participant and/or in participant-dependent changes in expression levels, not technical error (Supplementary Fig. 4). Importantly, normalization by the endogenous control miRNAs enables the comparison of changes in miRNA levels between participants and blood draws. Consequently, the calibrated-and-normalized data were used for all subsequent analysis.

### Longitudinal stability of MiRNA levels

**Figure 2:**
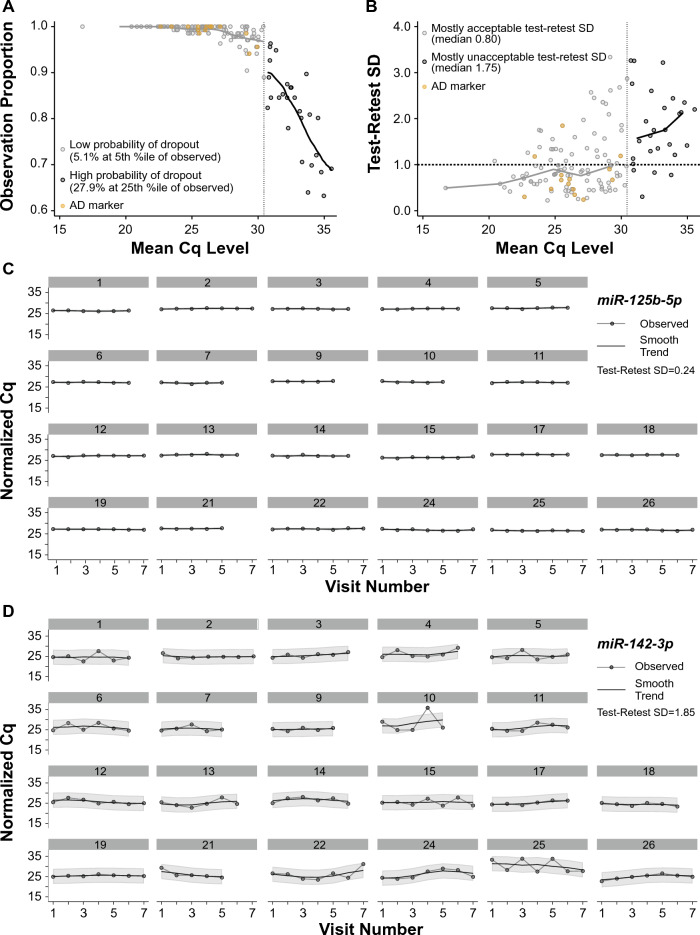
Lower Cq values increase the probability of miRNA amplification and stability. (**A**) For the 134 miRNAs that passed filtering (circles), at Cq < 30.44 (empirically-chosen threshold; see “Methods”) there is a low probability of dropout (only 5.1% dropout even at the low 5th percentile of miRNAs falling below the threshold, ordered by proportion observed), with 106 miRNAs having a high proportion of observation/amplification across all plasma samples (light grey and yellow circles). At Cq > 30.44 there is a high probability of dropout (27.9% dropout already at the higher 25th percentile of miRNAs falling above the threshold, ordered by proportion observed), and most of the miRNAs do not reliably amplify (28 miRNAs, dark grey circles). (**B**) For the 134 miRNAs that passed filtering, at Cq < 30.44 there are 70 miRNAs with acceptable test–retest standard deviation (SD) < 1.0 (light grey and yellow circles below dashed black line) and 36 miRNAs with test–retest SD > 1.0. At Cq > 30.44 there are 4 miRNAs with test–retest SD < 1.0 and 24 miRNAs with test–retest SD > 1.0 (dark grey circles). (**A**,**B**) MiRNAs we previously identified as candidate biomarkers for AD in CSF are depicted by yellow circles. (**C**,**D**) Calibrated and normalized Cq values within each participant (n = 22) and across each visit number are shown for a (**C**) stable (miR-125b-5p) and (**D**) unstable (miR-142-3p) miRNA.

A primary goal of the analysis was to identify miRNAs that have stable levels across time in plasma of healthy human adults of moderate age. Thus, miRNAs that are never or almost never observed in human plasma as measured by qPCR, or that are difficult to measure in repeated assessments, are inherently “unstable” (at least in terms of measurability) and cannot be meaningfully scored along a quantitative stability index scale; sparse miRNAs of this sort are not acceptable as biomarkers. For the 134 miRNAs that passed filtering and were included in the stability and metadata analysis (Supplementary Table 1A) we first assessed the impact of qPCR Cq value on the probability of observing a good amplification that meets quality control (QC) requirements (Fig. 2A), and on the within-participant standard deviation (SD) over test–retest (Fig. 2B). We use test–retest SD to assess the time-stable intraindividual variability of the Cq measurements and to provide information regarding the effect sizes needed to detect differences between clinical populations using our qPCR protocols and analysis pipeline; miRNAs with a lower test–retest SD are more sensitive to small effect sizes between groups. For miRNAs with mean Cq values < 30.44 (an empirically-chosen cutoff; see “Statistical analysis of stability” in “Methods”) we observe a low probability that a qPCR reaction will not reliably amplify (Fig. 2A). We find 74 miRNAs with acceptable test–retest SD < 1.0, 70 of which are at mean Cq < 30.44, including 11 of the miRNAs we previously identified as biomarkers for AD (Fig. 2B). As an example of a stable miRNA that is also a candidate AD biomarker, we show the calibrated and normalized Cq values for miR-125b-5p. Within each participant and across visits, miR-125b-5p had good within-participant reliability with a Cq value of ~ 26 and a test–retest SD = 0.24, which is well within the acceptable range (Fig. 2C). Interestingly, even though miR-142-3p, another candidate AD biomarker, also had a Cq of ~ 26, this miRNA had poor intraindividual stability as evidenced by a test–retest SD = 1.85 (Fig. 2D).

**Figure 3:**
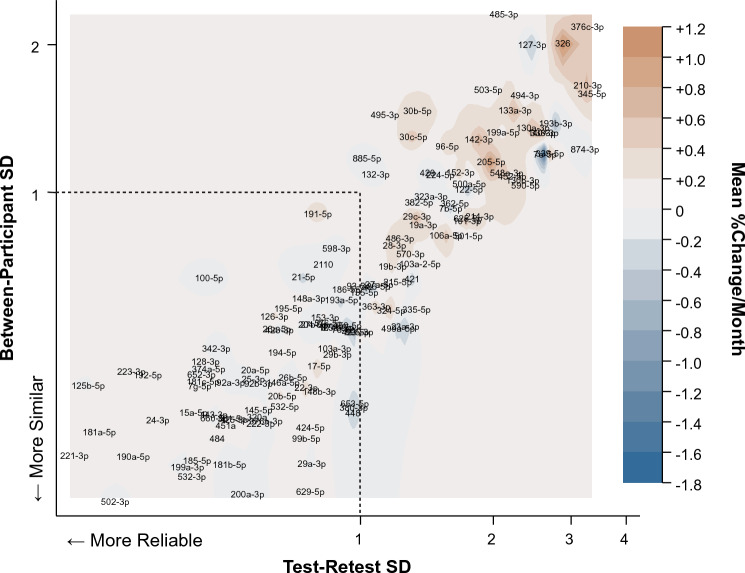
Indexing the stability of plasma miRNAs based on the intraindividual and interindividual variance and longitudinal drift. The 134 miRNAs that passed filtering were analyzed and plotted on a contour plot based on the between-participant SD (y-axis), the intraindividual test–retest SD (x-axis), and longitudinal drift that was quantified as the mean percent change in expression/month (color contours, intensity indicating a larger amount of drift). SD = 1.0 is set as the cutoff for acceptable variance (demarcated by dashed lines) along each axis, with 74 miRNAs having both between-participant and test–retest SDs < 1.0.

To obtain a more comprehensive assessment of the 134 miRNAs we analyzed each based on intraindividual test–retest SD, between-participant SD, and the longitudinal drift of miRNA levels in plasma as a percentage change from the initial value per month (mean % change/month) (Fig. 3 and Supplementary Table 1A). More reliable time-stable miRNAs are associated with lower test–retest SDs, which implies any single measurement will have better accuracy in estimating the plasma levels in a participant. MiRNAs with lower between-participant SDs have plasma levels that tend to be similar in all healthy participants, regardless of their biological characteristics (e.g., sex, age). Our data show that of the 134 miRNAs included in the stability analysis, 74 (55.2%) had both a test–retest and between-participant SD < 1.0 (Fig. 3, bottom left quadrant). Furthermore, these 74 miRNAs also showed less longitudinal drift as evidenced by smaller deviations from 0 in terms of the mean % change/month, compared to miRNAs with SDs > 1.0 (Fig. 3, color contours).

### Impact of hemolysis and participant metadata on MiRNA expression and variance

**Figure 4:**
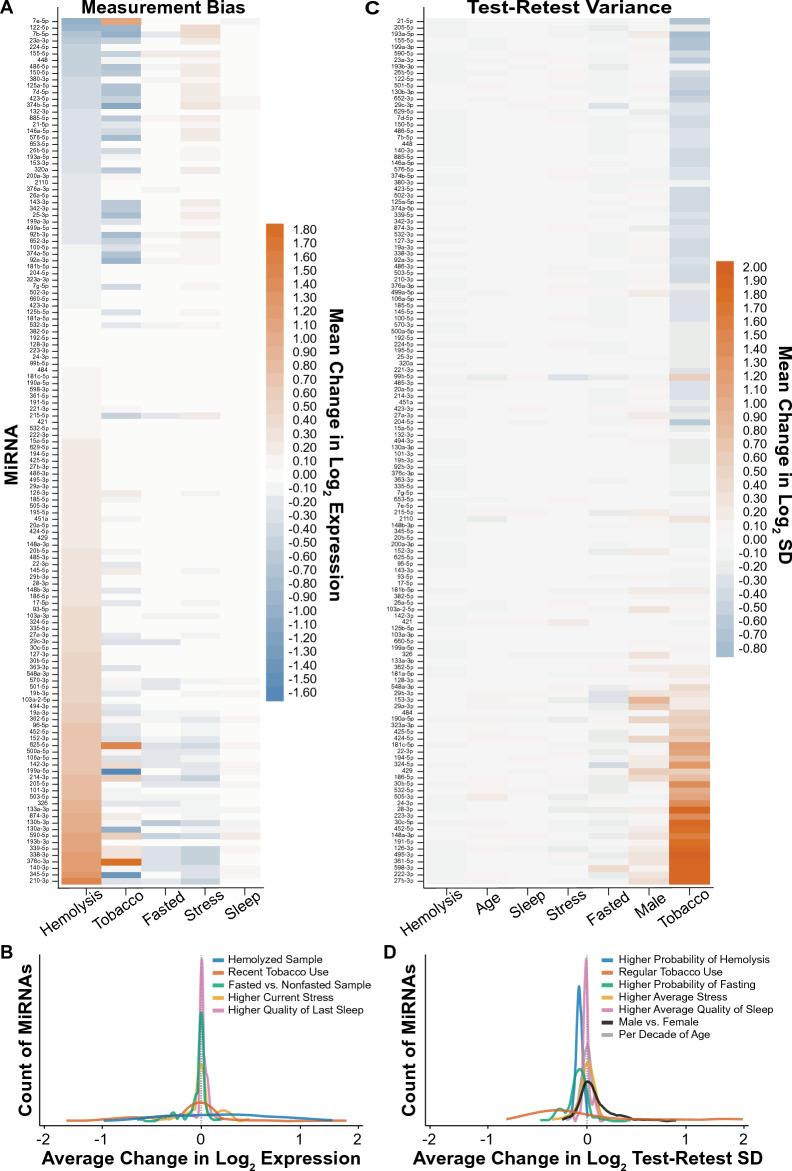
Hemolysis and tobacco use are factors that broadly impact plasma miRNA stability. (**A**) Measurement bias (mean deviation in log_2_ expression away from the predicted longitudinal trend) in the intraindividual expression levels for each of the 134 miRNAs that passed filtering, as associated with hemolysis of the plasma, self-reported measures of tobacco use, fasting status, higher stress level, and higher quality of last sleep. (**B**) Kernel density representation of the count distribution of the miRNAs from panel (**A**) relative to the average change in log_2_ expression, by associated factor. (**C**) Test–retest variance moderation (mean deviation in log_2_ SD magnitude away from the sample-average value) for each of the 134 miRNAs that passed filtering, as associated with hemolysis of the plasma, participant age per decade of life, higher average quality of sleep over the sampling window, higher average stress, higher probability of fasting, participant sex, and regular tobacco use. (**D**) Kernel density representation of the count distribution of the miRNAs from panel (**C**) relative to the average change in log_2_ test–retest SD, by associated factor. Analyses for each factor included adjustments for all the other factors (e.g., hemolysis results for measurement bias are adjusted for tobacco, fasting, stress, and sleep).

Next, for the 134 miRNAs we sought to demonstrate the impacts of hemolysis, self-reported measures of tobacco use, fasting status, higher stress level compared to prior visit, and change in the quality of sleep compared to prior visit on the intraindividual levels for each miRNA (Fig. 4A,B, Supplementary Table 1C). Note that data shown for each factor included adjustments for all the other factors (e.g., hemolysis results are adjusted for tobacco, fasting, stress, and sleep); see “Methods” for details. Note that sex and age of participant are not applicable factors to consider as influences on measurement bias, which quantifies the amount of within-subject change in miRNA levels caused by a factor being different at one occasion vs. at another occasion for the same donor; since sex and age are time-stable across the 3-month context of this study. By design they cannot differ from one occasion to another and thus cannot cause changes in miRNA levels between visits. Our data demonstrates that hemolysis of the plasma was related to excess variance in level changes for a majority of miRNAs (Fig. 4A,B, blue line), with 27 miRNAs showing at least a 1.5 fold increase (log_2_(1.5) = 0.58) and 4 miRNAs with 1.5 fold decrease in plasma levels per unit increase in hemolysis score. We also demonstrate that recent tobacco use had a large impact on the levels of 22 miRNAs (± 1.5 fold change), while fasting status, change in stress level, and change in sleep quality had minimal impact on miRNA levels; we observed no miRNAs with > 1.5 fold change due to these factors (Fig. 4A,B). We then examined the impact of average degree of hemolysis (across visits for the same participant), participant age per decade of life, higher average quality of sleep, higher average stress, higher probability of fasting, sex of participant, and regular tobacco use on the intraindividual test–retest SD (Fig. 4C,D, Supplementary Table 1D). We demonstrate that the magnitude of test–retest variance of most miRNAs was unaffected by a majority of the factors including age and average sleep quality, stress, and fasting probability (Fig. 4C,D). However, there was a large impact of regular tobacco use on the test–retest variance with 25 miRNAs showing a 1.5 fold increase in variance and 4 miRNAs with 1.5 fold decrease (Fig. 4C,D, orange line). The participant’s sex also sometimes impacted the test–retest SD as evidenced by > 1.5 fold increases in variance magnitude in males for 3 miRNAs (Fig. 4C,D).

## Discussion

The goals of this study were to identify the intraindividual longitudinal stability of miRNAs in human plasma and to identify common factors that impact miRNA levels and/or variance. We performed qPCR for 375 miRNAs on platelet free plasma (PFP) from 22 participants collected every 2 weeks for up to 16 weeks total (8 blood draws). We identified a set of 134 miRNAs that had an acceptable amplification in at least 63% of the visits for a participant, including 36 miRNAs that amplified in 100% of the samples (Supplementary Table 1A). Our studies also assessed the intraindividual stability and between-participant variance of the 134 miRNAs and found that 74 miRNAs (55.2%) had acceptable variance in day-to-day blood draws within a participant and blood collected from different participants (Fig. 3, SD < 1.0 on both axes). Furthermore, these 74 miRNAs had minimal longitudinal drift, compared to miRNAs with greater variance (Fig. 3, color contours). We also demonstrate that hemolysis and recent tobacco use had the greatest impact on fluctuations intraindividual miRNA levels (Fig. 4A) among the factors that we tested. Regular tobacco use also most greatly impacted intraindividual variance (Fig. 4C). Together, these data provide needed information pertaining to the reliability of plasma miRNAs that have potential utility as biomarkers for human diseases.

A key feature of valid human biomarkers is the reliability and constancy of measurements within and between healthy humans that enables changes resulting from a disease to be discernable. In line with this, we compared the detection rates of the 36 miRNAs we observed in 100% of the plasma samples herein to seven prior publications that also assayed miRNAs in plasma and/or serum of healthy humans^29–32,36–38^ (Table 1). Note that one publication assayed plasma and serum using both TaqMan and Exiqon miRNA assays^38^, (Table 1, denoted as “T” and “E”). Thirty-four of the 36 miRNAs were previously reported in human blood by at least one of the prior publications, and miR-223-3p, 451a, and 92-3p were identified in six publications, including by both the TaqMan and Exiqon assays (Table 1). Further, 21 of the 36 miRNAs were also detected in a 2012 study, in 100% of their plasma samples (n = 6) using TaqMan arrays^38^ (Table 1; “T”). Another study assessed the intraindividual stability of plasma miRNAs in 10 humans at four time points (days 1, 2, 7, 10), and identified a different set of 21 of the 36 miRNAs in at least 80% of their plasma samples^32^ (Table 1). Furthermore, they show that five of these miRNAs (miR-223-3p, miR-17-5p, miR-24-3p, miR-20a-5p, miR-146a-5p) do not have significant day-to-day variance^32^, which we corroborate with test–retest SDs < 1.0 (Table 1). While these studies demonstrate good reliability of assessing miRNAs in human plasma, the differences in detection rates across studies are likely attributed to different methods used to process blood samples and isolate RNA, miRNA analysis platforms (e.g., TaqMan vs. Exiqon), quality control metrics for miRNA exclusion/filtering, and data analysis procedures. Thus, future studies in larger cohorts of participants with consistent methodologies are needed to more completely define the plasma miRNAs that may serve as biomarkers for human disease.

**Table 1:** List of 36 miRNAs always detected in plasma samples and their values for intraindividual test–retest standard deviation (SD), between-participant (B-P) SD, longitudinal drift (mean % change/month), and human plasma and/or serum references. Notes: Wang^38^ assays were performed using TaqMan (T) and Exiqon (E); Wu^30^ detection rates in 50% or 90% of plasma samples are denoted.

MiRNA	Test–retest SD	B-P SD	Drift	Plasma or serum references
221-3p	0.22	0.29	0.09	30 (50%), 32, 36
125b-5p	0.24	0.41	− 0.01	36, 38 (T)
181a-5p	0.26	0.33	0.04	32, 36, 37
502-3p	0.28	0.24	− 0.03	36, 37
223-3p	0.30	0.43	0.14	29, 30 (90%), 32, 36, 37, 38 (E, T)
24-3p	0.35	0.35	0.14	32, 36, 37, 38 (E, T)
199a-3p	0.40	0.28	− 0.08	30 (50%), 32, 36, 37, 38 (E, T)
15a-5p	0.42	0.36	0.12	36, 37, 38 (E)
185-5p	0.42	0.29	0.16	29, 30 (50%), 36, 37, 38 (E, T)
652-3p	0.43	0.43	− 0.09	32, 36, 37, 38 (E, T)
let-7g-5p	0.43	0.41	− 0.05	30 (50%), 32, 36, 37, 38 (E, T)
128-3p	0.44	0.45	0.12	32
181c-5p	0.44	0.41	0.20	None
484	0.47	0.32	0.13	30 (50%), 36, 37, 38 (E, T)
143-3p	0.47	0.35	− 0.14	32, 36
342-3p	0.47	0.48	− 0.07	31, 36, 37, 38 (E, T)
660-5p	0.47	0.35	0.01	37, 38 (T)
451a	0.49	0.34	0.22	29, 30 (90%), 32, 36, 37, 38 (E, T)
92a-3p	0.51	0.41	− 0.08	29, 30 (90%), 32, 36, 37, 38 (E, T)
425-5p	0.52	0.35	0.21	30 (50%), 36, 37, 38 (E)
25-3p	0.57	0.42	− 0.13	30 (90%), 32, 36, 37, 38 (E, T)
145-5p	0.58	0.36	0.19	36, 38 (E, T)
20a-5p	0.58	0.44	0.15	30 (50%), 32, 36, 37, 38 (E, T)
320a	0.58	0.35	− 0.23	29 32, 36, 37, 38 (E, T)
92b-3p	0.59	0.41	− 0.17	29
146a-5p	0.67	0.41	− 0.18	30 (50%), 32, 36, 37, 38 (E, T)
26b-5p	0.70	0.42	− 0.20	32, 36, 37, 38 (E)
22-3p	0.75	0.40	0.32	30 (90%), 36, 37, 38 (E)
17-5p	0.80	0.44	0.39	30 (50%), 32, 36, 37, 38 (E, T)
125a-5p	0.87	0.53	− 0.21	36, 38 (E, T)
374b-5p	0.88	0.53	− 0.19	32, 36, 37
let-7d-5p	0.91	0.53	− 0.24	32, 36, 37
150-5p	0.93	0.54	− 0.30	32, 36, 38 (E, T)
448	0.96	0.36	− 0.29	None
155-5p	1.02	0.63	− 0.27	31, 32, 38 (T)
486-5p	1.08	0.64	− 0.40	29, 36, 37, 38 (E)

Another factor that may impact the outcome of miRNA biomarkers is whether there is a need for the enrichment of extracellular vesicles (EVs) prior to miRNA quantification. EVs are membrane-bound spheres that are secreted from a cell through either the endosomal pathway (exosomes) or direct budding from the plasma membrane (microvesicles)^39,40^. MiRNAs are selectively sorted into EVs by RNA-binding proteins such as hnRNPA2B1^41,42^. In addition to EVs, smaller nanoparticles (e.g., exomeres, supermeres) present in plasma show very high expression of specific miRNAs and miRNA-binding proteins^25^. Thus, EVs, exomeres, and supermeres have distinct miRNA profiles that can be enriched relative to the cellular profile^25,43^. As the miRNA cargo of EVs can be altered by pathological processes there is great interest in their potential as disease biomarkers^42,44–46^. However, different methods currently used for the enrichment of EVs and small nanoparticles from biofluids result in varying degrees of separation from non-EV particles. Thus, comparing the outcome of results between studies utilizing different enrichment strategies is challenging.

Our studies demonstrate that certain factors have a greater impact on miRNA measurement stability than others, with hemolysis having the greatest impact on fluctuations in intraindividual miRNA measurements (Fig. 4). It is well established that hemolysis and the release of miRNAs from red blood cells greatly alters the measured level of many miRNAs detected in plasma and serum^33,36,47–49^. Consequently, analysis methods have been developed to correct for this potential confound in blood-based miRNA studies^33,36,47^. Here we used the difference in Cq values of miR-23a-3p (insensitive to hemolysis) and miR-451a (sensitive to hemolysis) as a proxy measure of hemolysis severity^36,47^. In our 136 blood draws, we identified appreciable hemolysis (scores > 7) in about 25% of the samples and the other 75% of samples all showed varying degrees of hemolysis. In addition, recent tobacco use also greatly impacted intraindividual miRNA levels and the test–retest variance of many miRNAs (Fig. 4), consistent with our prior studies reporting differential levels of plasma EV miRNAs between smokers and non-smokers^50^. In contrast, one study reported that, with the exception of miR-128-3p, current healthy smokers (median packs/year = 46.3) had no significant difference in serum miRNA levels compared to healthy non-smokers^33^; note that we did not address the question of differential levels, only whether reliability of miRNA measurement may differ between smokers and non-smokers. However, the same study did report that there were no significant effects of fasting status on serum miRNA levels^33^, which was consistent with the findings in this study (Fig. 4). Another study reported that there was no significant impact of either fasting or sex on plasma miRNAs levels^32^, consistent with findings from this study that sex generally does not impact the within-subject variance of most miRNAs, with the exception of perhaps a few miRNAs that appeared to have somewhat larger test–retest variance in males (Fig. 4C,D). Participant age also did not generally affect the within-subject variance of plasma miRNAs assayed over the course of the study (Fig. 4C,D), and thus could not cause measurement bias from one visit to the next for the same donor because not enough time was allowed to pass between visits. Together, these studies and ours demonstrate the need to account and adjust for certain potential confounding factors on blood miRNA levels (such as hemolysis), while also reassuring that typical assays, such as qPCR, are robust to some commonly varying factors (e.g., fasting status, stress levels). In addition, the example of tobacco shows that the impact of other confounding factors such as drugs or alcohol use must be considered for miRNAs under consideration as disease biomarkers.

Relevant to our previous studies on candidate miRNA biomarkers for AD in CSF^14,26^, there is the potential for select miRNAs to serve as longitudinal disease biomarkers, such as the miRNAs that showed decreasing expression across the ordered diagnosis of control to MCI to AD^27^. However, CSF collection is an invasive procedure relative to blood, and requires specialized training and tools. Thus, translation of CSF miRNA biomarkers to blood would greatly improve the utility of clinical assays for central nervous system diseases. For example, our studies herein revealed that 14 candidate AD miRNAs in CSF were expressed in plasma, and 11 of these show acceptable intraindividual stability (Fig. 2B, test–retest SD < 1.0) and between-participant variance (SD < 1.0). Further, three miRNAs that trend in expression from control to MCI to AD in CSF^27^ are also expressed in the plasma samples evaluated herein. Relevant to our findings, a combination of miR-146a-5p, 181a-5p, and 148a-3p has been proposed as a blood-based assay for diagnosing age-related cognitive impairment and monitoring MCI progression to AD^51^. MiR-146a-5p expression is detected in plasma and/or serum studies in healthy human adults^30,32,36–38^, including two studies that detected expression of this miRNA in at least 80% of the samples^32,38^. Here we detected miR-146a-5p in 100% of the plasma samples assayed, as well as 181a-5p and 148a-3p in 100% and 95% of the plasma samples, respectively. These miRNAs also have acceptable intraindividual and between-subject variance, with miR-181a-5p having the third lowest test–retest variance (Supplementary Table 1A).

We also show the longitudinal expression of two published AD-associated CSF miRNAs as an example of candidate biomarkers for a neurological disorder: miR-125b-5p (Fig. 2C) shows stable expression over the 3-month time period, while miR-142-3p (Fig. 2D) shows variable expression over the same time period. These results demonstrate that some promising miRNAs, such as miR-142-3p, show greater variance over time in control participants, and thus may require a larger effect size in order to reliably signal differences in diseases such as AD, especially if decisions would be made using measurements taken at a single point in time. For single assays, a more reliable miRNA such as miR-125b-3p may be preferred if it is also clinically useful, but even noisy assays can generally be improved by repeating and averaging measurements. So variable expression over time does not negate the biomarker potential of miRNAs. For example, a 2.5 fold decrease in miR-142-3p expression has been reported in AD plasma compared to controls^52^, and on the log scale this corresponds ~ 0.7 × the test–retest SD and ~ 1.0 × the between-participant SD measured for this miRNA in our study, representing a sizeable expected effect size of ~ 0.6 (i.e., log_2_(2.5)/√(1.8^2^ + 1.3^2^) = 0.6) in a comparison involving healthy controls similar to ours. It is important to reiterate that the stability studies herein were assessed in healthy control participants; the stability of miRNAs may improve within a diseased population if the disease state is the primary driver for expression.

Relevant to the biology of miRNA biomarkers of disease are their effects on mRNA targets. Predicted mRNA targets for both miR-125b-5p and miR-142-3p are associated with pathways relevant to many human diseases. Top pathways for miR-125b-5p predicted gene targets include cellular senescence and apoptosis signaling, and within the apoptosis signaling pathway two predicted targets of miR-125b-5p are BCL2 and BCL2 Antagonist/Killer 1 (BAK1). BCL2 and apoptosis are associated with AD^53^, but studies investigating the effect of miR-125b-5p on BCL2 are still needed. However, in cortical neurons treated with amyloid-β peptide, miR-125b-5p is increased and BAK1 proteins levels are decreased^54^. The top predicted pathways for miR-142-3p include molecular mechanisms of cancer, cardiac hypertrophy signaling, and protein kinase A signaling, and one target for miR-142-3p is RAC1, which is implicated in cancer, viral infection, and in brain and myocardium ischemic injury^55–60^. RAC1 is a Rho-GTPase that is involved in axon growth, dendritic branching, and spine formation, and has been implicated as a therapeutic target of AD^61^. Further, RAC1 expression in human AD frontal cortex is decreased, but RAC1 is increased in AD plasma^62^. Yet studies investigating miR-142-3p regulation of RAC1 in AD are lacking.

Studies performed with small numbers of miRNAs support that plasma miRNAs are relatively stable following both short- and long-term storage at − 80 °C. For example, eight miRNAs analyzed from five healthy human participants showed a high stability and long frozen half-life (6–12 months, out to 14 years) in plasma^63^. A separate study on eight miRNAs from 10 healthy human participants showed that circulating miRNAs are stable for decades of storage at ultra-low temperatures and several freeze–thaw cycles^64^. And unlike mRNAs, miRNAs can resist digestion by ribonucleases because of their association with core components of silencing complexes such as Argonaute proteins, and/or by chemical modifications such as methylation^65^. Thus, our study herein on miRNAs in plasma stored for 7–10 months at − 80 °C represents an example of a relatively short period of time in storage, and our generally high recovery and stable measurement of dozens of known plasma miRNAs supports that the miRNAs are not degraded, in line with findings that plasma miRNAs are stable for years and even decades at − 80 °C. However, a number of pre-analytical variables can impact miRNA levels in blood, including phlebotomy procedures, centrifugation, filtration, RNase inhibitors, platelet removal, RNA isolation methods, and qPCR platforms^66–69^, and variations in plasma preparation and miRNA purification can account for up to 73% of the total intra-assay variance^68^. Our study attempted to mitigate technical causes for differences in plasma RNA levels by (i) performing all blood draws at one institute; (ii) using a single standardized protocol for plasma preparation; and (iii) controlling for time in storage by isolating plasma RNA within a relatively short storage time (7–11 months post collection) relative to the length in storage in a typical biomarker bank.

In conclusion, prior studies demonstrate that blood-based miRNAs can be reliably measured in healthy humans. We identified a set of 134 miRNAs that are reliably detected in healthy human plasma, 74 of which show longitudinal stability both within and between healthy adult participants. However, future studies that include a large number of participants and detailed attention to technical and biological variations are still needed to fully evaluate and identify stable miRNAs in blood in order to prove their clinical utility as biomarkers and to establish standardized clinical assays for specific diseases.

## Methods

### Participant samples

All procedures were approved by the Institutional Review Board (IRB) of the Oregon Health and Science University (OHSU IRB 22177). All human research participants provided written informed consent. Twenty-six healthy participants were recruited to donate blood under fasting conditions every other week for at least 7 donations over the course of 16 weeks. Four participants did not complete the study protocol, which resulted in a final cohort of 22 participants that donated blood up to 8 times. Some participants consented to a maximum of 7 blood draws (n = 8, black Supplementary Fig. 1) or missed a scheduled blood draw (n = 5, yellow Supplementary Fig. 1). To reduce technical variance our goal was to process all plasma samples using standardized protocols for plasma collection, preparation of PFP, and miRNA assays. Because of these criteria some blood draws were omitted due to a necessary change in a centrifuge used to prepare the PFP (n = 26, red Supplementary Fig. 1). One randomly-selected sample was also omitted due to a limited stock of the TaqMan Advanced miRNA Human A Cards from matching lot numbers (n = 1, blue Supplementary Fig. 1). This resulted in a total of 136 blood draws that were analyzed with the following distribution across participants: n = 4 with 5 draws, n = 10 with 6 draws, and n = 8 with 7 draws (Supplementary Fig. 1).

### Participant data

Metadata for each participant included sex, age, time of blood draw, and current medications. Prior to each blood collection, participants were surveyed regarding self-reported measures of (i) fasting status (‘time of last meal’), (ii) stress (‘are you stressed’: yes/no), (iii) sleep quality for the night prior to blood draw (scaled rating: 1 extremely poor, 2 poor, 3 average, 4 good, 5 extremely good), (iv) recent tobacco use (packs/day), and (v) other drug use (cannabis, alcohol). Additionally, females were asked (i) the date of last menstrual cycle, (ii) use of birth control, (iii) form of birth control, and (iv) anything else that they felt may affect hormone levels. A cursory assessment to investigate potential effects due to current medications (“neuroactive” vs “other” vs “none”), other drug use (yes/no for any alcohol use or any cannabis use), and female hormonal changes (time since last menses, use of any birth control, or any perceived hormonal difference) was performed and these factors were found not to overtly impact miRNA expression or variance. However, due to the limited sample size and the wide range of responses, especially concerning the types and amounts of current medications, the study was not powered sufficiently to include these factors into the statistical analysis for the metadata. Thus, conclusions from this analysis do not take into consideration current medications, drug use other than tobacco, or female hormonal changes.

### Plasma collection

Blood samples were collected by venipuncture into 10 mL draw capacity BD Vacutainer tubes containing 18 mg of K_2_EDTA (366643, BD Vacutainer EDTA, Becton, Dickinson and Company, Franklin Lakes, NJ). Participants were asked to provide fasting blood donations, and most did (Supplementary Fig. 2B). Following collection, tubes were stored upright at room temperature for at least 30 min until centrifugation within 1 h of collection and processing to obtain PFP. Briefly, blood was centrifuged at room temperature (22–25 °C) for 10 min at 450×*g* in an Allegra 6 centrifuge (Beckman Coulter, Inc., Pasadena, CA). The plasma layer was removed without disturbing the interface and pipetted into a fresh 15 mL Falcon tube. The plasma was then centrifuged for 15 min at 2500×*g* in an Eppendorf 5702 centrifuge (Eppendorf, Hamburg, Germany) and the supernatant transferred to a new 15 mL Falcon tube. This centrifugation process was repeated once more. The resulting PFP was transferred to 1.5 mL Eppendorf tubes in 500 μL aliquots, the aliquots were flash frozen on dry ice and stored at − 80 °C until RNA isolation.

### RNA isolation

Total RNA was isolated from 200 μL of PFP plasma using the miRNeasy Serum/Plasma Advanced Kit (217204, Qiagen, Hilden, Germany) following the manufacturer’s protocol. Plasma samples from an individual participant were grouped and processed together to mitigate within-participant RNA isolation variability and qPCR batch effects. Following the addition of lysis buffer to the plasma sample, an exogenous cel-miR-39-3p spike-in control (Thermo Fisher, Waltham, MA) was added at a final concentration of 3 pM. RNA was eluted from the column with 20 μL of nuclease free water. The miRNA concentration of each sample was measured using the Qubit miRNA Assay kit (Thermo Fisher) and read using a Qubit 4.0 fluorometer (Thermo Fisher).

### MiRNA qPCR profiling

MiRNA was converted to cDNA using the TaqMan Advanced miRNA cDNA Synthesis kit (A28007, Thermo Fisher). Briefly, 3 μL of total RNA was 3′ poly-adenylated, followed by a 5′ adaptor ligation step and reverse transcription. The resulting cDNA (5 μL) was added to a 14 cycle universal miR-amplification step. The miR-amplification reaction was diluted 1:10 with nuclease free water, then 220 µL of the diluted cDNA was mixed with 220 µL of nuclease free water and 440 µL of TaqMan Fast Advanced master mix (4444556, Thermo Fisher). The qPCR mix (100 µL) was loaded into each of 8 ports in a TaqMan Advanced miRNA Human A Card (A34714, Thermo Fisher). The miRNA Human A card has 384 wells and includes assays for 375 human miRNAs, a primary endogenous normalization control (miR-16-5p, n = 5 technical replicates), an exogenous spike-in calibration control (cel-39-3p, n = 2 technical replicates), and exogenous negative control (ath-miR159a, n = 2 technical replicates). Each card was assayed using a QuantStudio™ 7 Flex Real-Time PCR System (4485695, Thermo Fisher) with the aid of an Orbitor RS2 Microplate Mover (ORB2001, Thermo Fisher) to increase throughput and ensure each participant’s time course samples were assayed on the same day. Each participant’s qPCR data was imported into ExpressionSuite Software version 1.3 (Thermo Fisher) for automatic baselining and thresholding, with maximum Cq set to 40 cycles. Then each participant’s data was exported from ExpressionSuite as one individual data file, resulting in 22 data files in total. For all subsequent analyses these 22 data files were imported and processed together using Stata version 17.0 software (StataCorp LLC, College Station, TX).

### MiRNA qPCR quality control metrics

The qPCR reactions were assessed by multiple QC metrics. A qPCR reaction was considered ‘acceptable’ if the AmpScore (signal rise in linear phase) was ≥ 1, and the CqConf (calculated confidence in the Cq value) was ≥ 0.8. For acceptable Cq values, we examined the appropriateness of the baseline separation from the recorded Cq value and compared the QC assessments with other qualitative flags exported by ExpressionSuite, finding nothing unusual. Acceptable Cq values < 34 were used as exact values in the analysis. qPCR assays that did not meet the acceptability criteria (or that were labeled “Undetermined” by ExpressionSuite) were considered ‘missing’ measurements, regardless of any recorded Cq value. Reactions with AmpScore ≥ 1.0 and CqConf ≥ 0.8, but with Cq values ≥ 34 were considered ‘censored’ values. Both missing and censored values were given special handling in the analysis (see “Cq normalization”). We also examined the exogenous negative control (ath-miR159a) reactions to verify that no sample had a Cq value recorded with acceptable QC. The exogenous spike-in positive control (cel-miR-39-3p) and primary endogenous normalization reference control (miR-16-5p) reactions were 100% observed with acceptable QC in all samples.

### Filtering of sparsely observed miRNAs

MiRNAs that are never or almost never observed in human plasma using qPCR profiling methods are not suitable as plasma biomarkers. Therefore, we excluded miRNAs that did not meet reasonable standards of feasibility of measurement based on the proportion of visits for a participant where the miRNA had acceptable Cq. The included miRNAs were those that showed the highest longitudinal coverage of visits for a donor across the most donors. This was achieved in an approximate way by selecting all miRNAs where average longitudinal coverage across donors was at least 63.2%. The cutoff corresponds to 1−1/*e*, the theoretical solution based on sequential selection by a greedy algorithm (which at every step would add a new miRNA to the set if the average coverage was improved by doing so, and improved more by adding that miRNA than by adding any other miRNA not still in the set), and will usually select the top *n* choices, where *n* is the total sample size. Of the 378 miRNAs (including controls) on the array card, we selected 136 miRNAs with average coverage exceeding the 63.2% cutoff. As cel-miR-39-3p and miR-16-5p controls were two of the 136 selected miRNAs, only 134 miRNAs of scientific interest were considered in the stability and metadata analysis (Supplementary Table 1A). The remaining 242 miRNAs were excluded from further analysis based on: (i) no amplifications in any sample (30 miRNAs), (ii) all amplifications failed QC (83 miRNAs), or (iii) most amplifications failed QC (129 miRNAs) (Supplementary Table 1B). Note that the mean longitudinal coverage for the excluded miRNAs was only 17% (median 9%), versus 92% (median 97%) for the miRNAs included in the stability and metadata analysis.

### Hemolysis proxy assessment

The difference in raw absolute Cq values of miR-23a-3p (insensitive to hemolysis) and miR-451a (sensitive to hemolysis) performs well as a proxy measure of hemolysis severity in serum^47^. Both miR-23a-3p and miR-451a showed acceptable longitudinal coverage and were selected for inclusion at the filtering step (“Filtering of sparsely observed miRNAs”). We calculated the hemolysis score (Cq for miR-23a-3p minus Cq for miR-451a) for each sample and compared it to our qualitative assessments of sample color and clarity, finding good agreement (e.g., the most visibly pinkish samples were scored as being most affected by hemolysis). The hemolysis score was then used as an adjustment variable in both the calibration and normalization.

### Cq calibration with exogenous cel-miR-39-3p spike-in control

For technical calibration all raw Cq values were adjusted for RNA isolation differences via median-alignment of an exogenous calibration factor. To generate an exogenous calibration factor based on a robust estimate of technical variance for each plasma sample, we averaged the mean Cq of the duplicate cel-miR-39-3p reactions and the median Cq across the 36 always-observed miRNAs (Supplementary Table 1A). We identified the always-observed miRNAs by scanning the 134 miRNAs that passed filtering (“Filtering of sparsely observed miRNAs”) with an acceptable Cq in every visit sample, both on a per-participant basis and across all participants. The median Cq value of these 36 miRNAs was calculated for each visit sample for each participant, and correlated at 0.89 (Spearman rank correlation) with the mean of the duplicate cel-miR-39-3p wells in the array. The samples were median-aligned to one another using this exogenous calibration factor prior to batch calibration and endogenous normalization.

### Cq batch calibration

Batch calibration attempts to remove residual technical artifacts in the Cq measurements attributable to processing order (i.e., the run sequence of plasma samples in the qPCR assays). We first centered all cel-miR-39-3p spike-in calibrated Cq values for the 134 miRNAs by subtracting the mean (across visits) for the participant for each miRNA and then fit a flexible smooth (nonlinear) curve to the centered values across the processing sequence using nonparametric kernel regression with smoothing bandwidth determined by cross-validation. We then subtracted this curve from the input Cq values and next fit a mixed-effects model to the kernel regression residuals using REML (restricted maximum likelihood) estimation and conditioning on the acceptability of the reaction (acceptable, missing, censored), hemolysis score (entered as a quadratic effect to allow for nonlinearity of impact of hemolysis on Cq residuals within a batch, e.g., if batches contained clusters of high-hemolysis and low-hemolysis samples in varying and very unequal proportions), and other technical flags (e.g., whether the distance between the Cq value and the baseline was larger than 8 cycles or not, etc.). We used this model to estimate the technical impact of RNA isolation batch (date and time of day) as random effects via the estimated BLUPs (best linear unbiased predictions). The BLUP values were then subtracted from the sequence-adjusted Cq values as batch effects. The resulting sequence-and-batch-corrected Cq values were then recentered to the global median of the input Cq value distribution.

### Selection of endogenous miRNAs for normalization

In addition to mean levels of miR-16-5p (n = 5 technical replicates per array card), two additional sets of endogenous miRNAs were used to normalize endogenous levels of the miRNAs across participants (set 1) and across visits within each participant (set 2). Set 1 comprised two miRNAs (miR-24-3p, miR-484) selected from among the 36 always-observed miRNAs and was used for interparticipant normalization. Set 2 included 5 miRNAs (miR-103a-3p, miR-126-3p, miR-191-5p, miR-30e-5p, miR-93-5p) where at least a subset of the 5 was consistently observed in every visit sample for any given participant, but not necessarily the same subset for all participants, so the exact subset of miRNAs used was allowed to differ by participant; this set was used to normalize across visits uniquely for each participant. MiRNAs from both sets have also been recommended by Thermo Fisher Scientific for endogenous normalization based on stable expression in serum and plasma (i.e., miR-24-3p, mir-484, miR-126-3p, miR-191-5p, miR-93-5p), as well as miR-16-5p (https://assets.thermofisher.com/TFS-Assets/GSD/Reference-Materials/identifying-mirna-normalizers-white-paper.pdf). The median Cq values of miRNAs in each set respectively for each visit sample were separately incorporated into the Cq normalization model.

### Cq normalization

After the sequential cel-miR-39-3p spike-in calibration and batch calibration, we performed an endogenous normalization using a multilevel interval regression model that accounts for censored and missing Cq values, as well as longitudinal correlation in the observed Cq values across visits for the same participant. Noncensored Cq values with acceptable QC were used as exact measurements. Censored and missing Cq values at a given visit were handled within the model by imputing the most-likely value consistent with both the normalizer prediction and values observed for the miRNA in other visits for the same participant. For censored Cq values, the imputed Cq value fell in the range between the calibrated Cq value for the miRNA and the maximum observable Cq value (Cq = 40). For missing Cq values, the imputed Cq value was any value > 40. Thus, each visit sample was given a definite predicted Cq value and a standard error associated with that prediction based on the variance not explained by the model. This standard error was propagated to downstream analyses to account for imputation error. The multilevel interval regression model was fit on each miRNA individually, including fixed effects of the (i) mean for miR-16-5p at the visit, (ii) median of miR-24-3p and miR-484 (always-observed miRNAs; set 1), (iii) median of up to 5 participant-specific miRNAs (set 2), and (iv) hemolysis score for the sample at the visit, to be aware of biased predictions based on hemolyzed samples. All Cq values for the miRNA for each participant at each visit were included in the normalization model, with random effects of participant to account for residual correlation in the Cq values across visits for each participant. Participant-specific visit-level predictions from the miRNA-specific models were used as the normalized miRNA Cq values. After this final normalization step, the resulting values were adjusted for RNA isolation and qPCR efficiency differences, batch effects associate with different qPCR runs, differences in stable endogenous background level between participants, as well as visit-to-visit differences in stable endogenous background level within participants.

### Statistical analysis of stability

Analysis of the fully-normalized Cq values was carried out miRNA-by-miRNA. For each of the 134 miRNAs, we fit a within-subjects linear regression of the normalized Cq value on the visit sequence, clustering by participant. This considered the full set of visits for a single participant as a block, and assessed the trend in visits over time only for that participant. We then averaged the trends across the participants. A cluster-robust sandwich (Huber-White) estimator was used for variance assessments of the time trend. The following statistics were then collected from the above regression model for each miRNA: (i) the grand mean across participants, to compare miRNAs to each other by level; (ii) the percentage change over time to assess average longitudinal drift in the Cq values (a stable marker should have 0 drift on average); (iii) the total residual variance to assess “noisiness” of the miRNA on average (lower is better); (iv) the portion of residual variance explained by participant differences to assess miRNA level variance across a healthy adult population; and most importantly, (v) the unexplained portion of residual variance, which is the key estimate of test–retest SD when considering hypothetical retest at the same occasion in a random participant. A visual analysis of observation proportions and longitudinal stability statistics by mean Cq (Fig. 2) suggested a qualitative break in performance between miRNAs with average levels above vs. below Cq = ~ 30, such that higher-concentration miRNAs have better measurement and reliability properties. Thus, we used a nonparametric method (supremum testing over all possible breakpoints) to empirically estimate the breakpoint (mean Cq) associated with the sharpest distinction between measurement dropout rates before vs. after the break. This analysis estimated Cq = 30.44 as a threshold for comparing typical performance between miRNAs with mean Cq levels on opposite sides of the threshold.

### Statistical analysis of metadata

Metadata analyses (Fig. 4) were based on ridge regression, a type of machine learning also known as regularized regression. We used ridge regression because of the somewhat large number of predictors (5 for measurement bias, 7 for test–retest variance regressions) relative to the number of observations (n = 22, representing an average support of 3 or 4 observations per predictor). In ordinary regression techniques, the estimates with large sampling variance may be mostly hidden from the model and misfit to model assumptions when observation counts within predictor combinations are too small to adequately support robust estimation of associations. Ridge regression techniques account for this by shrinking most coefficients to negligible amounts and preserving impactful coefficient magnitudes for only the predictor combinations with the most robust assumptions suggested by the data. The regression weights of the predictors are shrunk towards zero using a quadratic penalty. Note that all predictor coefficients are shrunk, but in a way that low-information combinations are shrunk more than high-information combinations. This is an example of a bias-variance tradeoff where model parameters incur a certain amount of nullward bias in exchange for stabler estimation with lower expected sampling variance. Moreover, all effects are automatically adjusted for all other effects included in the model, but with lower effective degrees of freedom (i.e., the adjustments are partial, depending on the relative shrinkage amounts). For example, measurement bias regressions in this study included hemolysis, tobacco use, fasting status, stress, and sleep quality, so the effects for each one were partially adjusted for all the others, and thus represent best-guess estimates of the impact of each included factor on the measurement bias and test–retest SD when the other factors are approximately controlled for.

Ridge regressions were performed separately for measurement bias (i.e., the visit-level error for a participant at a particular visit after adjusting for any time trend across visits; Fig. 4A) and test–retest variance (i.e., the typical error magnitude for a participant across all visits; Fig. 4C). The ridge regressions for measurement bias included only time-variable predictors that could differ in value from visit to visit for a participant (e.g., sleep quality the night before the visit). The ridge regressions for test–retest variance included only time-invariant (e.g., sex) and time-averaged factors (e.g., average sleep quality across all visits for the participant). A fivefold cross-validation was used to select the optimal ridge penalty parameter. Importantly, *each* ridge regression (the separate regression for *each* miRNA on *each* of the two stability outcomes) is associated with a different penalty parameter. The magnitude of each penalty depends on (i) the predictor scales and associations for the miRNA for the outcome; and (ii) the miRNA-specific scaling of the outcome itself. Thus, the penalty values cannot be meaningfully compared across different regressions, except in the sense that smaller penalties (closer to zero) more closely approximate ordinary (unregularized) least-squares linear regression. The heatmaps in Fig. 4 show the directions (color hue) and magnitudes (color intensity) of regression weights from the ridge regressions.

### Supplementary Information

Supplementary Information.

Supplementary Tables.

## Data Availability

All data used for statistical comparisons in this study, the amplification flag summary by miRNA and experimental group used to select miRNAs for differential expression or differential detection, and the datasets generated for this study are included in the Gene Expression Omnibus (GEO; https://www.ncbi.nlm.nih.gov/geo/).
