# Supplementary material for: Analysis of the longitudinal stability of human plasma miRNAs and implications for disease biomarkers
Source: Sci Rep. 2024 Jan 25;14:2148. doi: 10.1038/s41598-024-52681-5 (PMC10810819; doi:10.1038/s41598-024-52681-5)
Supplement: Supplementary file 1 — Supplementary Information. [file 41598_2024_52681_MOESM1_ESM.docx]

**Analysis of the Longitudinal Stability of Human Plasma MiRNAs and Implications for Disease Biomarkers**

**SUPPLEMENTARY FIGURES 1-4**

**Supplementary Figures Legends**

**Supplementary Figure 1. Participant Plasma Samples.** Twenty-two healthy participants donated blood up to 8 times. A total of 136 blood draws were analyzed with the following distribution across participants: n=4 with 5 draws, n=10 with 6 draws, and n=8 with 7 draws. Missing data were the result of *i*) participants consenting to a maximum of 7 blood draws (n=8, black); *ii*) participant missing a scheduled blood draw (n=5, yellow); *iii*) omission due to inconsistent processing during platelet free plasma preparation (n=26, red); or *iv*) or omission due to limited stock of miRNA array cards (n=1, blue).

**Supplementary Figure 2. Distribution of Participant Age and Fasting Status for the Blood Draws. (A)** Age distribution of male (n=7) and female (n=15) participants binned into four-year increments. **(B)** Distribution of the number of blood draws relative to the hours since last meal (Fasting Duration). Samples categorized as “Fasted” with fasting duration > 8 hours or “Not Fasted” with fasting duration < 8 hours.

**Supplementary Figure 3. Distribution of Hemolysis Scores for the Plasma Samples.** The hemolysis score for each plasma sample was calculated as: ∆Cq = miR-23a-3p (insensitive to hemolysis) - miR-451a (sensitive to hemolysis). Plasma samples were categorized by low (< 5), moderate (5 - 7), or severe (> 7) score, with a majority of the plasma samples being in the low to moderate range (to the left of the dashed line at 7).

**Supplementary Figure 4. Correcting MiRNA qPCR Assay Variance with Sequential Calibrations.** Residuals by miRNA and participant were calculated for the **(A)** raw Cq values of all 136 miRNAs included in the analysis (grey solid line); **(B)** the Cq values following calibration by cel-miR-39-3p (black short dashed line); **(C)** the Cq values following sequential calibration by cel-miR-39-3p then batch and run sequence (black long dashed line); and **(D)** the calibrated (cel-39-3p and batch) Cq values following normalization to endogenous control miRNAs (solid black line). The residuals for each miRNA were centered around 0 by subtraction of the mean level (across all samples) within each miRNA, and by subtraction of the mean level (across all visits) within each donor.

**Supplementary Figure 1.
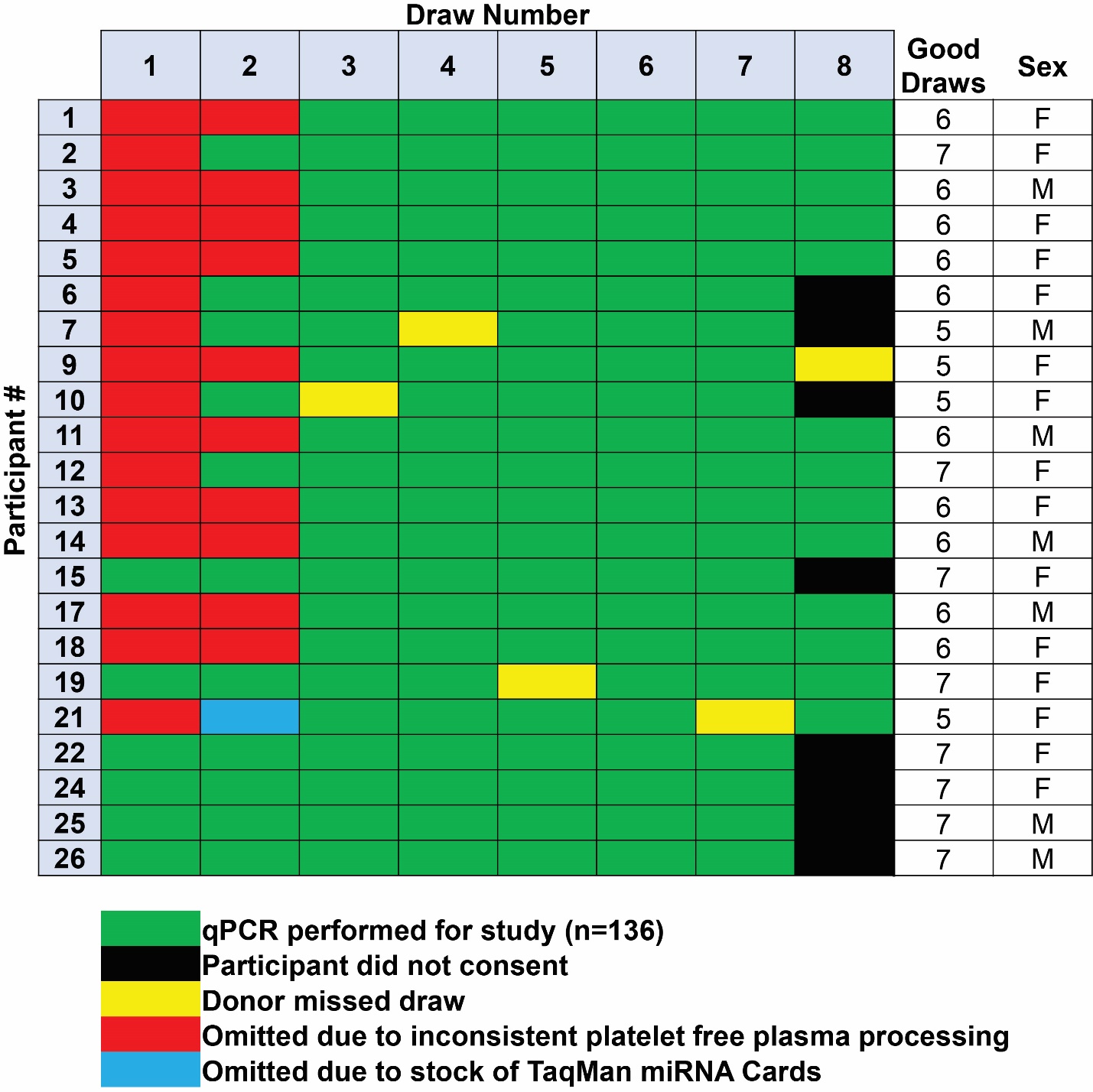
**

**Supplementary Figure 2.**


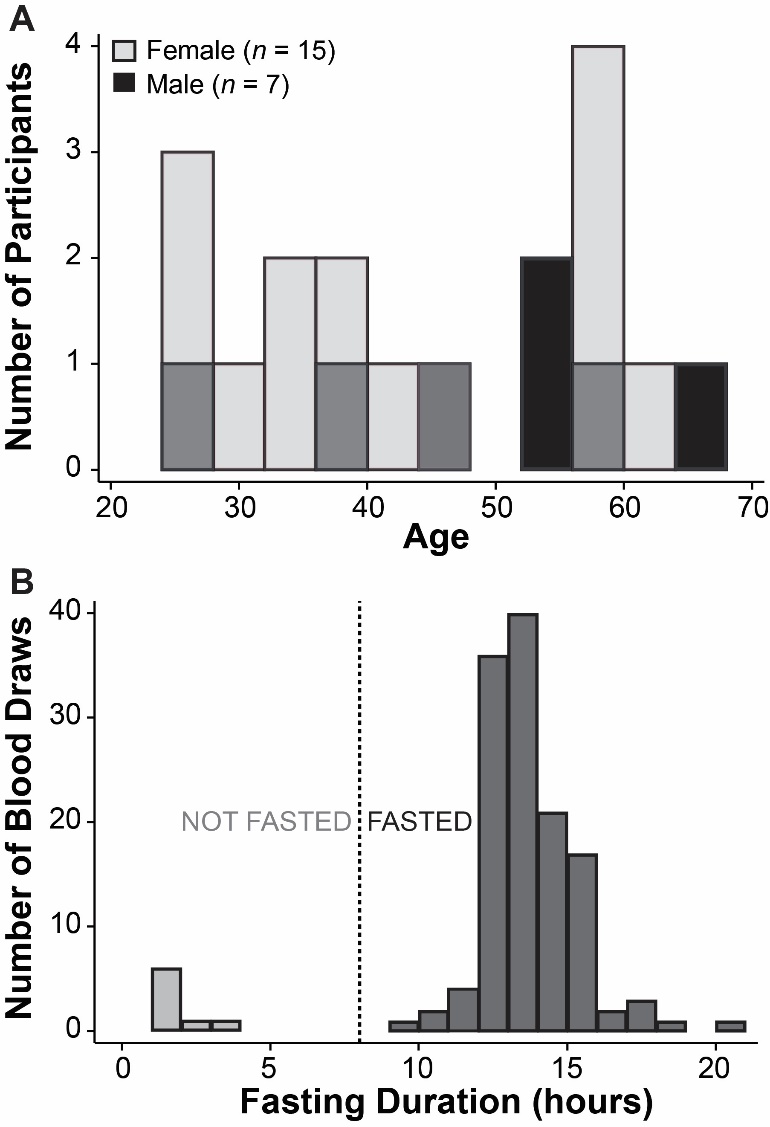


**Supplementary Figure 3.**

**
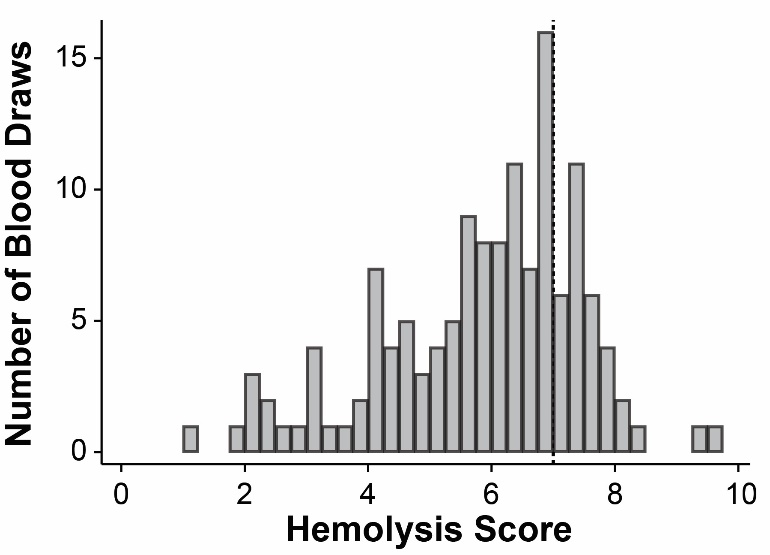
**

**Supplementary Figure 4.**


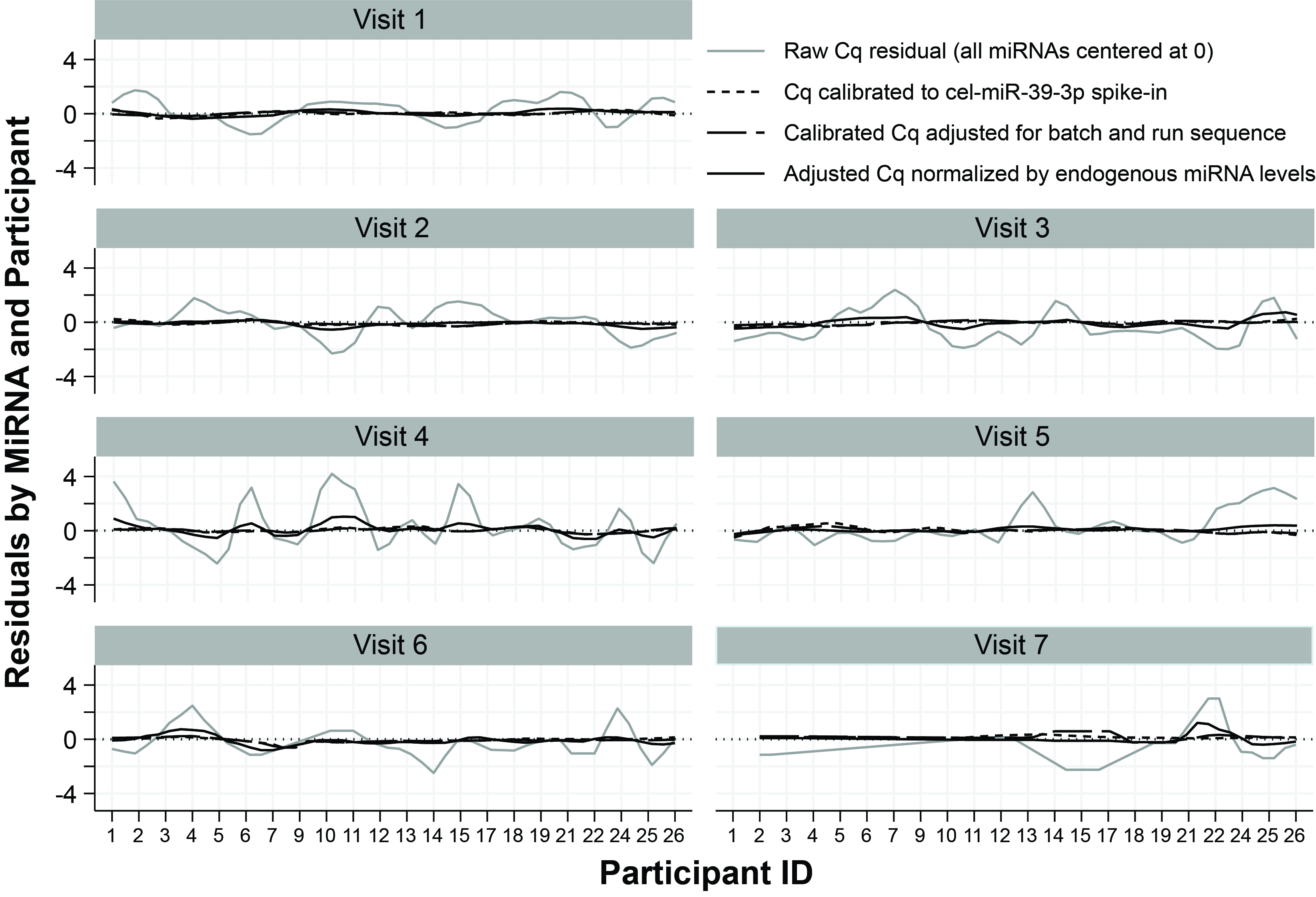


**SUPPLEMENTARY TABLE 1A-D LEGENDS**

**Supplementary Table 1A. Assay Variance on MiRNA Raw Cq Values in Participant Plasma Samples.** Assay variance on miRNA raw Cq values revealed a set of 36 miRNAs that are always observed within each participant, on each study visit date. Table 1A includes: **a**. MiRNA; **b**. Mean % participant where always observed = 100%; **c**. % Total Quantifications; **d**. Mean Cq Level; **e**. Intraindividual Test-Retest SD; **e**. Between-Participant SD; **g**. Mean % Change/Month.

**Supplementary Table 1B. MiRNAs Excluded from Further Analysis.** 242 remaining miRNAs were excluded from further analysis. Table 1B includes: **a**. MiRNA; **b**. Exclusion Criteria; **c**. Mean % Participant Where Always Observed; **d**. % Amplifications Passing QC; **a**. Mean % Visits Acceptable. Table 1B column **b**. Exclusion Criteria includes: *i*) most amplifications failed QC (129 miRNAs), *ii*) all amplifications failed QC (83 miRNAs, and *iii*) no amplifications in any sample (30 miRNAs).

**Supplementary Table 1C. Measurement Bias of Factors that Broadly Impact Plasma MiRNA Stability.** Measurement bias (mean deviation in log_2_ expression away from the predicted longitudinal trend) in the intraindividual expression levels for each of the 134 miRNAs that passed filtering. Table 1C includes: **a**. MiRNA; **b**. Plasma Hemolysis; **c**. Tobacco Use; **d**. Fasting Status; **e**. Higher Stress Level; **f**. Sleep Quality.

**Supplementary Table 1D. Test-retest Variance Moderation.** Test-retest variance moderation (mean deviation in log_2_ SD magnitude away from the sample-average value) for each of the 134 miRNAs that passed filtering. Table 1D includes: **a**. MiRNA; **b**. Plasma Hemolysis; **c**. Per Decade of Age; **d**. Sleep Quality; **e**. Higher Stress Level; **f**. Fasting Status; **g**. Male vs. Female; **g**. Tobacco Use.
